# Surgery

**Published:** 1894-12

**Authors:** 


### Progress in Medical Science.

### SURGERY.

### DOUBLE FEMORAL HERNIOTOMY IN A WOMAN 64 YEARS OF AGE; PRIMARY UNION OF BOTH WOUNDS.

Dr. S. E. Milliken, New York, (*La Revista Med. Quirurg,*) reports a case of double femoral herniotomy at the advanced age of 64 years. Deep sutures of kangaroo tendon were used to close the crural canal, while catgut was employed for bringing together the skin wounds. The dressings were changed for the first time on the tenth day, when union was found complete, and the superficial sutures had been absorbed. The highest elevation of temperature was 101° F., which occurred within forty-eight hours, and was attributed to the shock of the operation.

CONCLUSIONS.

1. Age is no contra-indication to the employment of the radical cure of hernia.

2. Asepsis and antisepsis should be carefully observed.

3. Even in cases of strangulation, the radical cure should be attempted, if the condition of the patient warrants the delay.

4. When the truss becomes a source of annoyance, or if the hernia is difficult to retain, the operation should be performed without delay, and before strangulation occurs.

36 West 59th Street.

